# Bed site selection by a subordinate predator: an example with the cougar (*Puma concolor*) in the Greater Yellowstone Ecosystem

**DOI:** 10.7717/peerj.4010

**Published:** 2017-11-14

**Authors:** Anna Kusler, L. Mark Elbroch, Howard Quigley, Melissa Grigione

**Affiliations:** 1 Department of Biology, Pace University, Pleasantville, NY, United States of America; 2 Panthera, New York, NY, United States of America

**Keywords:** Cougar, *Puma concolor*, Bed site, Refugia

## Abstract

As technology has improved, our ability to study cryptic animal behavior has increased. Bed site selection is one such example. Among prey species, bed site selection provides thermoregulatory benefits and mitigates predation risk, and may directly influence survival. We conducted research to test whether a subordinate carnivore also selected beds with similar characteristics in an ecosystem supporting a multi-species guild of competing predators. We employed a model comparison approach in which we tested whether cougar (*Puma concolor*) bed site attributes supported the thermoregulatory versus the predator avoidance hypotheses, or exhibited characteristics supporting both hypotheses. Between 2012–2016, we investigated 599 cougar bed sites in the Greater Yellowstone Ecosystem and examined attributes at two scales: the landscape (second-order, *n* = 599) and the microsite (fourth order, *n* = 140). At the landscape scale, cougars selected bed sites in winter that supported both the thermoregulatory and predator avoidance hypotheses: bed sites were on steeper slopes but at lower elevations, closer to the forest edge, away from sagebrush and meadow habitat types, and on southern, eastern, and western-facing slopes. In the summer, bed attributes supported the predator avoidance hypothesis over the thermoregulation hypothesis: beds were closer to forest edges, away from sagebrush and meadow habitat classes, and on steeper slopes. At the microsite scale, cougar bed attributes in both the winter and summer supported both the predator avoidance and thermoregulatory hypotheses: they selected bed sites with high canopy cover, high vegetative concealment, and in a rugged habitat class characterized by cliff bands and talus fields. We found that just like prey species, a subordinate predator selected bed sites that facilitated both thermoregulatory and anti-predator functions. In conclusion, we believe that measuring bed site attributes may provide a novel means of measuring the use of refugia by subordinate predators, and ultimately provide new insights into the habitat requirements and energetics of subordinate carnivores.

## Introduction

Interspecific competition contributes to the structure of ecological communities, including species assemblages (Gotelli & McCabe, 2002). In systems with multiple competing carnivores, these species must contend with exploitative and interference competition for resources, as well as direct and indirect threats such as harassment, kleptoparasitism, and interspecific killing (Durant, 1998; Palomares & Caro, 1999; Durant, 2000; Creel, Spong & Creel, 2001; Odden, Wegge & Fredriksen, 2010; Vanak et al., 2013; Lendrum et al., 2014; Elbroch et al., 2015a). In such systems, dominant competitors can exclude or limit subordinate competitors (MacArthur & Levins, 1967); thus, subordinate predators must balance energy expenditures associated with collecting critical resources with the costs associated with interactions with more dominant competitors (Creel, Spong & Creel, 2001; Vanak et al., 2013; Elbroch et al., 2015b). Such pressure directly influences resource selection by subordinate predators at numerous scales, from the microsite (Elbroch et al., 2015c), to the home range (Broekhuis et al., 2013; Vanak et al., 2013; Lendrum et al., 2014), to the greater landscape (Gotelli & McCabe, 2002; Berger & Gese, 2007).

Historically, many studies of resource selection were limited to coarse scale analyses due to the limitations of available technology (Lyons, Gaines & Servheen, 2003), or were restricted to species that were easily observable (e.g., Loveridge et al., 2006). With the emergence and increasing affordability of Global Position System (GPS) technology, however, it has been possible to assess behavioral decisions and resource selection of cryptic species by examining spatially aggregated GPS locations, termed “clusters” (Anderson & Lindzey, 2003). When visited in the field, the locations of clusters reveal information regarding prey killed by study animals (e.g., Anderson & Lindzey, 2003) or other behaviors of interest, such as parturition sites or winter hibernacula (e.g., Evans et al., 2012; Elbroch et al., 2015b). As technology has improved, the number of and rate at which GPS locations are taken has increased, thereby allowing researchers to examine resource selection at finer spatial and temporal scales. Such advances provide opportunities to explore previously understudied aspects of animal behavior, such as documenting locations where cryptic animals sleep.

Sleeping is among an animal’s most vulnerable behavioral states, and bed sites are an important ecological resource for many species (e.g., Chen et al., 1999; Germaine, Germaine & Boe, 2004; Linnell, Nilsen & Andersen, 2004). Though there are numerous hypotheses for what drives bed site selection, the predation avoidance (Hamilton, 1982; Linnell et al., 1999; Linnell, Nilsen & Andersen, 2004) and thermoregulatory (Lang & Gates, 1985; Millspaugh et al., 1998; Tull, Krausman & Steidl, 2001) hypotheses have garnered the most support (Savagian & Fernandez-Duque, 2017). The predation avoidance hypothesis posits that bed site selection reduces the likelihood of being killed by a predator or competitor (Messier & Barrette, 1985; Smith, Oveson & Pritchett, 1986; Mysterud & Ostbye, 1995), and research suggests that bed site selection among primates and ruminant ungulates is driven primarily by decisions that minimize predation risk (Anderson, 1998; Ramakrishnan & Coss, 2001; Lima et al., 2005; Singhal & Johnson, 2007) and increase survivorship (Canon & Bryant, 1997; Brodie & Brockelman, 2009; Grovenburg et al., 2010). Bed sites that minimize predation risk are often inaccessible to predators and/or offer high visual concealment (e.g., Matsuda, Tuuga & Higashi, 2010). The thermoregulatory hypothesis predicts that bed site selection aids in body temperature regulation (Anderson, 1998). Bed sites that promote thermoregulation typically maximize sun exposure in the coolest months and offer shade in the warmer months (Savagian & Fernandez-Duque, 2017).

Despite the importance of sleep or rest in mammals (Siegel, 2008), most research on bed site selection has been limited to primates and ruminant ungulates (e.g., Hamilton, 1982; Armstrong, Euler & Racey, 1983; Brodie & Brockelman, 2009). There has been significantly less research attention paid to bed selection by carnivores, for which bed sites may function as ‘competition refuges’ that allow subordinate predators to coexist with dominant species in natural landscapes (Durant, 1998). To date, we are aware of only four studies conducted on bed site selection by three carnivore species: wolves (*Canis lupus*) (Wam, Eldegard & Hjeljord, 2012), European lynx (*Lynx lynx*) (Sunde, Stener & Kvam, 1998), and cougars (*Puma concolor)* (Akenson, Henjum & Craddock, 1996; Akenson et al., 2003). In their respective study sites, both the wolf and European lynx were considered “apex predators,” species with no other non-human predators. Researchers examined the influence of human disturbance on the bed site selection of these two species, and found that even apex predators will select bed sites with high vegetative concealment to provide safety from perceived threats (Sunde, Stener & Kvam, 1998; Wam, Eldegard & Hjeljord, 2012).

The two remaining studies (Akenson, Henjum & Craddock, 1996; Akenson et al., 2003) examined bed site selection by cougars, a subordinate predator. Cougars are a large, solitary felid and the most widespread terrestrial carnivore in the western hemisphere (Sunquist & Sunquist, 2002). In the Greater Yellowstone Ecosystem (GYE) of North America, they are subordinate to wolves, American black bears (*Ursus americanus*), and grizzly bears (*Ursus arctos horribilis*). Bears primarily influence cougar kill rates by kleptoparasitizing cougar kills and forcing cougars to abandon prey they have killed (Murphy et al., 1998; Elbroch et al., 2015c). Wolves, however, have a strong influence on numerous aspects of cougar behavior and survivorship: wolves influence cougar prey selection (Kortello, Hurd & Murray, 2007; Elbroch et al., 2015a) and space use (Ruth, 2004; Kortello, Hurd & Murray, 2007; Lendrum et al., 2014), and directly kill cougars (Ruth, 2004; Kortello, Hurd & Murray, 2007; Elbroch et al., 2015a). In the southern GYE, wolves are the primary cause of mortality for cougar kittens <6 months old (Elbroch and Quigley, unpublished data), and research has demonstrated decreased cougar survival rates following the reintroduction of wolves (e.g., Kortello, Hurd & Murray, 2007). Early research of cougar bed site selection was relatively limited in scope. But in these studies, in which cougars were sympatric with black bears, researchers found that cougars chose bed sites with high vegetative concealment and that were close to escape terrain, defined as forested terrain with rim rock cliff structures and downed logs (Akenson, Henjum & Craddock, 1996; Akenson et al., 2003). Such characteristics suggest that cougars may indeed select bed sites that promote predator avoidance.

Our research expands upon these findings to test whether cougars selected bed sites that provided thermoregulatory benefits and/or mitigated predation risk in an ecosystem supporting a multi-species guild of competing predators. We tracked cougars in the southern GYE from 2012 to 2016 and examined bed sites at two levels of resource selection: the landscape level (“second-order selection,” which reflects habitat or resource selection by an individual or population across a landscape (Johnson, 1980)), and the microsite level (“fourth-order selection,” which reflects behavioral decisions made within specific habitat types within an individual’s home range or territory (Johnson, 1980)). At the landscape level, we predicted bed sites would be located in structurally complex habitat types such as forests, situated on steeper slopes to facilitate protection or escape from intraguild predators, and seasonally located on southern slopes to maximize access to solar radiation. At the microsite level, we expected beds would be closer to escape terrain, that they would have higher canopy cover to offer protection from the elements, and that they would bed in areas with greater vegetative concealment to hide them from potential competitors. Ultimately, we hypothesized that bed sites would be spatially explicit, that their spatial attributes would vary with season, and that bed sites would be driven by both thermoregulatory and predator avoidance functions.

## Materials and Methods

### Ethics statement

Our capture protocols for cougars, as outlined in Elbroch et al. (2013), adhered to the guidelines outlined by the American Society of Mammalogists (Sikes & Gannon, 2011), and were approved by two independent Institutional Animal Care and Use Committees (IACUC): the Jackson IACUC (Protocol 027-10EGDBS-060210) and National Park Service IACUC (IMR_GRTE_Elbroch_Cougar_2013-2015). Every effort to ameliorate suffering of cougars was made, and no cougars were killed or sacrificed during capture events. Our study was carried out on the Bridger-Teton National Forest (United States Forest Service, USFS Authorization ID JAC760804), Grand Teton National Park (NPS Permit GRTE-2012-SCI-0067), and the National Elk Refuge (USFW permit NER12), with permission to handle cougars granted by the Wyoming Game and Fish Department (Permit 297).

### Study area

**Figure 1:**
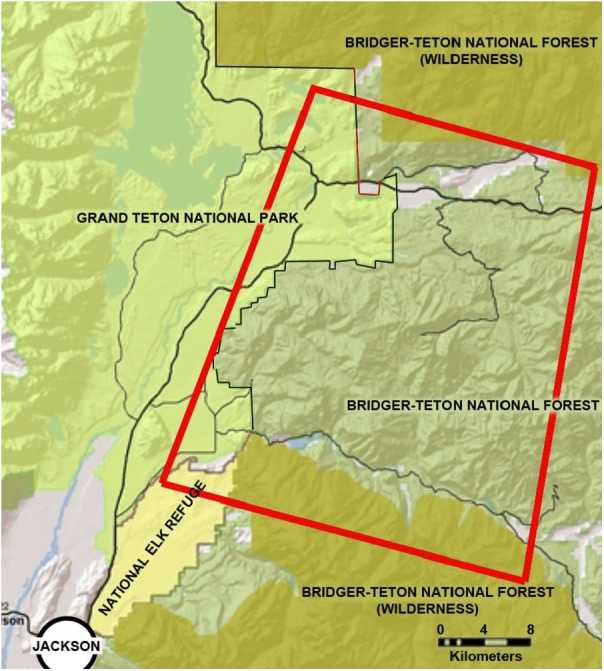
The location of the study area in northwestern Wyoming, USA. The study area is located northeast of the city of Jackson, Wyoming, and is delineated in this figure by a red line. It encompasses sections of the National Elk Refuge, Grand Teton National Park, and Bridger-Teton National Forest.

Our study area encompassed approximately 2,300 km^2^ of the southern Greater Yellowstone Ecosystem in Teton County, Wyoming, and included portions of the Bridger-Teton National Forest, Grand Teton National Park, and the National Elk Refuge (Fig. 1).

Elevations in the study area ranged from 1,800 m in the valleys to >3,600 m in the mountains. The area was characterized by short summers and long winters with frequent snowstorms. Average summer temperatures were 6.9 °C, and average winter temperatures were −7.2 °C (Gros Ventre SNOTEL weather station). Precipitation occurred mostly as snow, and maximum snow depths ranged from 100 cm at lower elevations to >245 cm at intermediate and higher elevations (2,000 m+). Habitats included foothill grasslands, big sagebrush (*Artemisia tridentate*) dominated shrub-steppe, Douglas-fir (*Pseudotsuga menziesii*) forests, aspen (*Populus tremuloides*) forests, and higher elevation coniferous forests, composed of lodge pole pine (*Pinus contorta*), subalpine fir (*Abies lasiocarpa*), Engelmann spruce (*Picea engelmannii*), and white bark pine (*Pinus albicaulis*). Riparian corridors were dominated by cottonwood (*Populus ungustifolia*, *Populus balsamifera*, and *Populus trichocarpa*) and willow (*Salix* spp.) communities (Marston & Anderson, 1991).

Other carnivores in the study system included grizzly bears, American black bears, wolves, coyotes (*Canis latrans*), and red foxes (*Vulpes vulpes*). Ungulate prey included elk (*Cervus elaphus*), mule deer (*Odocoileus hemionus*), white-tailed deer (*O. virginianus*), Shiras moose (*Alces alces shirasi*), bighorn sheep (*Ovis canadensis*), and North American pronghorn (*Antilocapra americana*).

### Cougar capture, collar programming, and bed site identification

Each winter, cougars were located, immobilized, and fitted with satellite GPS collars (Lotek Wireless, Inc.; Newmarket, Ontario, Canada; or Vectronic Aerospace GmbH., Berlin, Germany). We used trailing hounds to force cougars to retreat to a location where we could safely approach them. Cougars were immobilized with ketamine (4.0 mg/kg) and medetomidine (0.07 mg/kg), and their temperature, heart rate, and respiration were monitored at five-minute intervals while they were processed, sampled, and fitted with a collar. Once animals were completely processed, the effects of the capture drugs were reversed with Atipamezole (0.375 mg/kg), and cougars departed capture sites on their own.

We programmed collars to acquire location data between 12 and 24 times per day and received and uploaded data to Google Earth daily. We identified GPS clusters visually in Google Earth, which we defined as any ≥2 subsequent locations ≥4 h apart, within 150 m of each other, and occurring within two weeks of each other. Because of the marked temperature differences between winter and summer and the migratory behavior of cougar prey, we analyzed data for two seasons. Following Elbroch et al. (2013), we defined seasons based on well-established elk migrations: summer, defined as June 1–November 30, and winter, defined as December 1–May 31.

We visited and examined clusters in the field, and 98% of all investigations were performed by CyberTracker-certified observers (Evans et al., 2009; Elbroch et al., 2011), ensuring expertise and consistent field effort. Bed sites were identified as a circular depression in the vegetation or snow containing identifiable cougar hair. When consuming prey, cougars often bedded within the immediate vicinity of their kills. So, to ensure that our examination of bed site selection was distinct from kill site selection, we only included beds that were unassociated with known kills. Therefore our analysis included only those beds selected by cougars in between time periods associated with handling prey, and more than 500 m from confirmed kill sites.

### Second-order selection: comparing bed site attributes to landscape attributes

Following methods for resource selection functions (Boyce, 2006), we employed logistic regression with a binomial distribution (logit link function) to compare bed sites with random locations on the landscape; for each bed site, we sampled five random points from within the minimum convex polygon (MCP) bounding the location data of all our study animals during the study period, which we defined as our “study area.” Bed sites were mapped in ArcGIS 10.1 (ESRI, Redlands, CA, USA), and random points were generated using the Geospatial Modeling Environment (GME, Beyer, 2009–2012). Each bed site or random location was then assigned the following attributes: habitat type (*n* = 5), distance to forest edge, slope (30 m resolution), terrain ruggedness (a 3-dimensional vector ruggedness measure termed VRM (Sappington, Longshore & Thompson, 2007)), elevation, and aspect (categorized as north, south, east, and west).

We reclassified 87 land cover classes described in a Gap Analysis Program (gapanalysis.usgs.gov/gaplandcover) at 30 m resolution into five general habitat classes by lumping biologically similar cover classes together: (1) grasslands, meadow, or barren; (2) riparian and water bodies; (3) sagebrush and shrub-steppe; (4) forest; and (5) disturbed, agricultural, and urban. Aspect and VRM were derived from the digital-elevation model (http://datagateway.nrcs.usda.gov/) following the method of Sappington, Longshore & Thompson (2007). Forest edge was created following the methods of Elbroch et al. (2015b) by drawing a perimeter around each forested section.

To begin, we devised a list of bed site attributes that would support the thermoregulation hypothesis versus the predator avoidance hypothesis, from which we developed a set of *a priori* models to test against each other. We believed the following covariates influenced thermoregulatory properties of bed sites in both winter and summer: distance to forest edge, habitat type, aspect, and elevation. We believed that proximity to forest edges, aspect, and habitat class would modulate solar radiation and exposure to the sun or cold winds. Higher elevations in our study area were correlated with increased snowfall and lower temperatures, and thus we expected that in the winter, cougars would choose bed sites at lower elevations.

We believed the following covariates would influence predation risk by competitors at bed sites in both seasons: habitat class, proximity to forest edge, slope, and VRM. To mitigate predation risk, we expected cougars to select for more structurally complex habitat types and against open vegetation classes where cursorial wolves would have the advantage. We also expected beds to be closer to forest edges, to increase the likelihood of detecting approaching competitors. For example, research by Wam, Eldegard & Hjeljord (2012) found that wolves often bedded on “overlooking sites,” likely to facilitate the detection of conspecific intruders. We also believed that steeper slopes and more rugged terrain (high VRM) would increase bed site inaccessibility and/or facilitate escape from a potential predator.

Prior to any statistical analyses, we employed a correlation matrix to evaluate collinearity (|*r*| > 0.50) among predictor variables. Predictor variables were not correlated (all |*r*| < 0.50), so we included them all in our analyses. Cougar ID was included as a random intercept to account for variation among individuals. We then created candidate models for each season and hypothesis (winter thermoregulation, winter predator avoidance, summer thermoregulation, and summer predator avoidance) from all possible combinations of their distinctive, biologically-relevant predictor variables. We calculated Akaike’s Information Criterion adjusted for small sample size (AICc), ΔAICc, and Akaike weights (wi) for each model in each model set, and considered the top model and any subsequent model differing by <2 AIC_c_ units to have produced substantial empirical support for explaining variation in the data; redundant covariates in top models (e.g., nesting) were considered uninformative (Arnold, 2010), and when it occurred, we selected the simplest top model.

As models were constructed and analyzed in the same way across hypotheses and model sets, we also compared performance characteristics of our top thermoregulation and predator avoidance models, to see which best fit the data and which hypothesis garnered the most support from our data. Further, we ran an additional post-hoc analysis for each season, composed of a single model containing the significant parameters from each of the top thermoregulation and predation avoidance models, to determine if a combination of the two hypotheses performed better than either one alone (based upon AIC parameters).

### Fourth-order selection: microsite characteristics of bed sites

We calculated seasonal 95% fixed-kernel home ranges for each marked, resident adult cougar. Kernel density estimates (Worton, 1989; Kie et al., 2010) and isopleths were quantified in the Geospatial Modeling Environment (GME, Beyer, 2009–2012); for cougars sampled over multiple years, we calculated annual home ranges and then averaged parameters.

We collected microsite attributes at verified bed sites and 50 random points in each cougar’s seasonal home range (e.g., 50 points in their winter home range, and 50 in their summer home range). Following the methods of Elbroch et al. (2015b), we gathered the following microsite data: canopy cover, concealment, and habitat characteristics. Canopy cover was measured in each cardinal direction from the center of each location or bed with a convex spherical crown densiometer (Forestry Supplier, Kackson, MS, USA). Concealment was measured with a subdivided concealment board (Noon, 1981) measuring 1 m tall and 50 cm wide (Elbroch et al., 2015b). The concealment board was held at the center of each site, and we recorded the percent of the concealment board obscured by natural features when viewed from 10 m away in each cardinal direction. We also noted whether each site occurred on, under, or within one meter of a prominent physical feature such as a tree, cave, cliff band, boulder, or log jam. In comparison to our landscape-level analyses that employed a habitat class layer in ArcGIS sampled at a 30 m resolution, we also collected habitat and topography data at 10 m in each cardinal direction from the bed site. We defined the microhabitat of bed sites in the field as one of seven habitat types (forest, forest edge/transitional habitat, meadow, sagebrush, riparian, and a “rugged” barren habitat type consisting of cliff bands and talus fields) and the topography of each site as either a bench, cliff band, drainage, ridgeline, sloping hillside, or flat. Slope was derived in ArcGIS from the digital-elevation model (http://datagateway.nrcs.usda.gov/) at a resolution of 15 m. Finally, we also recorded distance to what we termed “escape terrain.” Initial research by Akenson, Henjum & Craddock (1996), Akenson et al. (2003) and preliminary field examinations in our study area demonstrated that many cougar beds were in or very near to rugged, structurally complex landscape features such as talus fields, cliff bands, and areas of downed woody debris. To test if they were selecting for proximity to these features, we used a range finder (Bushnell) to measure the distance from a bed to the nearest escape terrain feature, up to 200 m.

As with our landscape analysis, we first determined covariates that supported the thermoregulatory versus predator avoidance hypotheses. In both the winter and the summer, we included the following variables in our microsite selection models to support the thermoregulatory hypothesis: canopy cover, habitat class, and the presence of a physical feature such as a tree, cave, or boulder. As has been shown in cougar dens (e.g., Bleich et al., 1996), such covariates may provide protection from the elements, therefore assisting in body temperature regulation.

For both winter and summer, we included the following covariates to support the predator avoidance hypothesis of bed selection at the microsite level: concealment, slope, topography, habitat class, proximity to escape terrain, and the presence of a feature such as a tree or boulder. Vegetative concealment hides cougars from potential enemies, and proximity to escape terrain, structured habitats, steeper slopes, and physical features facilitate escape should a cougar be discovered in its bed.

We repeated the approach described above for our landscape-level analyses. We employed a correlation matrix to evaluate collinearity (|*r*| > 0.5) among continuous predictor variables and chi-square test of independence to test for collinearity between categorical predictors, but no predictor variables were correlated (all |*r*| < 0.50). Then, for each hypothesis and each season, we employed generalized linear mixed models with a binomial distribution (logit link function) and a random effect (intercept) to account for variation among individual cougars. For each hypothesis, we created a candidate model set derived from all possible combinations of the biologically-relevant covariates. We then calculated and compared AICc values, ΔAICc, and Akaike weights (wi) to determine whether the best predator avoidance model, the best thermoregulatory model, or the post-hoc combination model containing the significant parameters of both hypotheses best explained bed site selection at the microsite level.

## Results

### Second-order selection: comparing bed site attributes to landscape attributes

From 23 December 2012 to 19 January 2016 we visited 1,718 clusters. We documented 599 beds, 754 kills, and 366 sites where we did not find anything. The 599 beds were from nine different cougars (three males, six females). Of those, 312 were wintertime beds, and 287 were summertime beds.

In the wintertime, cougar bed selection supported both the thermoregulation and predator avoidance hypotheses (Table 1). The results from the post-hoc combination model showed that cougars selected beds in the winter that were at lower elevations (*p* < 0.001; *β* =  − 0.006), on steeper slopes (*p* < 0.001; *β* = 0.097), and closer to forest edges (*p* < 0.001; *β* =  − 0.008; mean distance: 57.29 m ± 54.99 m). They selected against sagebrush (*p* < 0.001; *β* =  − 1.299), meadow (*p* < 0.001; *β* =  − 1.355), and riparian habitat types (*p* = 0.005; *β* =  − 1.134). Though cougars did exhibit selection for eastern (*p* < 0.001; *β* = 1.375) and western aspects (*p* = 0.033; *β* = 0.514), selection was strongest for southern aspects (*p* < 0.001; *β* = 1.655).

**Table 1:** Top ranked model comparisons from the landscape-level logistic regression, including the number of parameters (K), the log-likelihood (logLik), AICc scores, ΔAICc, and model weight for landscape level selection; including aspect, elevation, slope, “edge” (distance to nearest forest edge), “VRM” (terrain ruggedness), and “veg” (habitat class).

Landscape level selection					
	K	logLik	AICc	ΔAIC	weight
**Winter**					
**Thermoregulation**					
aspect + elevation + edge + veg	4	−663.547	1351.3	0.00	1.000
aspect + elevation + edge	3	−695.572	1407.2	55.96	0.000
elevation + edge + veg	3	−705.185	1426.4	75.18	0.000
elevation + edge	2	−723.402	1454.8	103.56	0.000
aspect + elevation + veg	3	−719.971	1462.1	110.82	0.000
**Predator Avoidance**					
edge + slope + veg + VRM^a^	4	−749.385	1516.9	0.00	0.508
edge + slope + veg	3	−750.428	1516.9	0.07	0.492
edge + slope + VRM	3	−762.108	1534.2	17.38	0.000
edge + slope	2	−763.248	1534.5	17.65	0.000
slope + veg	2	−781.069	1576.2	59.33	0.000
**Thermoregulation + Predator Avoidance**					
aspect + elevation + edge + slope + veg	5	−591.900	1209.8	–	1.000
**Summer**					
**Thermoregulation**					
edge + veg	2	−746.243	1506.6	0.00	0.695
aspect + edge + veg	3	−743.029	1508.2	1.66	0.303
aspect + veg	2	−749.574	1519.3	12.72	0.001
veg	1	−753.973	1520.0	13.44	0.001
edge	1	−765.301	1536.6	30.06	0.000
**Predator Avoidance**					
edge + slope + veg	3	−691.872	1399.8	0.00	0.644
edge + slope + veg + VRM	4	−691.471	1401.0	1.22	0.351
slope + veg	2	−698.396	1410.9	11.03	0.003
slope + veg + VRM	3	−697.467	1411.0	11.19	0.002
edge + slope	2	−711.353	1430.7	30.90	0.000

**Notes.**

a Though this model was within 2 AIC units of the top model, VRM was an uninformative parameter (Arnold, 2010) and therefore the simpler model excluding VRM was determined the best model.

In the summertime, cougar bed site attributes strongly supported the predator avoidance hypothesis over the thermoregulation hypothesis (Table 1). We did not run an additional post-hoc combination model as the significant variables from the top thermoregulation model were already included within the top predator avoidance model. The top predator avoidance model found that cougars selected bed sites closer to forest edges (*p* < 0.001; *β* =  − 0.003; mean distance: 75.93 m ± 95.12 m) and on steeper slopes (*p* < 0.001; *β* = 0.077). They also selected against sagebrush (*p* < 0.001; *β* =  − 0.775) and meadow habitat types (*p* < 0.001; *β* =  − 1.333).

### Fourth-order selection: microsite characteristics of den sites

We assessed microsite attributes at 60 summertime beds (from six cougars) and 80 wintertime beds (from eight cougars). In the winter, bed site attributes supported the thermoregulation hypothesis over the predator avoidance hypothesis (Table 2). Based upon our top model, cougars disproportionately selected bed sites with high canopy cover (*p* < 0.001; *β* = 0.057; mean percent cover: 87.4% ± 22.9%) and in “rugged” barren habitats (*p* = 0.007; *β* = 3.048) characterized by cliff bands and talus fields. Selection for rugged habitat classes likely also mitigated predation risk. Though the presence of a feature such as tree or boulder was included in our top model (Table 2), the parameter was not statistically significant (*p* = 0.999).

**Table 2:** Top ranked model comparisons from the microsite-level logistic regression, including the number of parameters (K), the log-likelihood (logLik), AICc scores, ΔAICc, and model weight for landscape level selection; including slope, “veg” (habitat class), “canopy” (percent canopy cover), “conc” (percent vegetative concealment), “near_esc” (categorical variable denoting if a bed site was within 200 m of escape terrain), “on_feat” (categorical variable denoting if a bed site was on or under a physical terrain feature such as a tree or cliff), and “topo” (topography).

Microsite level selection					
	K	logLik	AICc	ΔAIC	weight
**Winter**					
**Thermoregulation**					
canopy + veg + on_feat	3	−98.197	214.8	0.00	1.00
canopy + on_feat	2	−112.417	232.9	18.14	0.00
canopy + veg	2	−120.858	258	43.24	0.00
veg + on_feat	2	−127.214	270.7	55.95	0.00
veg	1	−136.064	278.2	63.4	0.00
**Predator Avoidance**					
conc + slope + on_feat + near_esc	4	−110.106	232.4	0.00	0.401
conc + slope + on_feat	3	−111.391	232.9	0.52	0.309
conc + slope + on_feat + topo	4	−106.922	234.3	1.93	0.153
conc + slope + on_feat + topo + near_esc	5	−106.079	234.7	2.34	0.125
conc + slope + on_feat + near_esc + veg	5	−109.351	241.3	8.88	0.005
**Thermoregulation + Predator Avoidance**					
canopy + conc + slope + veg	4	−105.200	230.3	–	1.000
**Summer**					
**Thermoregulation**					
canopy + conc + on_feat	3	−98.028	206.2	0.00	0.587
canopy + conc	2	−100.078	208.3	2.05	0.211
canopy + conc + veg + on_feat	4	−94.364	209.3	3.09	0.125
canopy + conc + veg	3	−96.238	210.9	4.73	0.055
canopy + on_feat	2	−103.059	214.2	8.01	0.011
**Predator Avoidance**					
conc + on_feat + slope + topo	4	−101.754	224.1	0.00	0.293
conc + near_esc + on_feat + slope + topo	5	−100.762	224.2	0.13	0.275
conc + on_feat + topo	3	−102.972	224.4	0.33	0.249
conc + near_esc + on_feat _ topo	4	−102.559	225.7	1.61	0.131
conc + veg + on_feat + topo	4	−100.647	230.4	6.32	0.012
**Thermoregulation + Predator Avoidance**					
canopy + conc + topo + on_feat	4	−89.400	198.9	–	1.000

In the summer, cougar bed selection supported both the thermoregulation and predator avoidance hypotheses (Table 2). The results from the combination model showed that cougars selected bed sites with higher concealment (*p* = 0.002; *β* = 0.030; mean percent concealment: 82.2% ± 20.6%), higher canopy cover (*p* < 0.001; *β* = 0.053; mean percent canopy cover: 91.7% ± 17.2%), on steeper slopes (*p* = 0.004; *β* = 1.816), on benches (*p* = 0.002; *β* = 2.340), and in cliff band (*p* = 0.015; *β* = 2.440) topography.

## Discussion

We found strong support for our hypotheses that bed site selection by a subordinate apex predator supports thermoregulation and mitigates potential conflicts with competitors. Cougar bed site attributes varied with season and scale, reflecting different behavioral strategies to balance energy expenditures associated with resource acquisition with the potential costs of interactions with more dominant competitors. Our findings suggest refugia are a key resource for subordinate predators as well as prey species, and that greater research attention should be dedicated to this aspect of predator space use to complement existing literature that focuses on the effects of prey on predator distributions and movements (e.g., Litvaitis, Sherburne & Bissonette, 1986; Zabel, McKelvey & Ward Jr, 1995; Herfindal et al., 2005).

At the landscape level, the attributes of cougar beds reflected both thermoregulatory and predator avoidance functions. In the winter, cougars selected bed sites on south-facing aspects with increased sun-exposure. Southern aspects are an important wintertime landscape feature for many ungulate prey species in the northern hemisphere (e.g., Armstrong, Euler & Racey, 1983; Stewart et al., 2010). Cougars also selected against open habitat types such as sagebrush and meadows, which lack cover or complex structures to facilitate escape (Ruth et al., 2011), and where cursorial wolves were likely to have the advantage (Husseman et al., 2003). Further, cougars bedded on steeper slopes nearer to forest edges in winter, characteristics that supported escape from rather than confrontation with approaching competitors. This is particularly important for cougars in our study system, which experienced increased negative interactions with dominant wolves in the winter (Elbroch et al., 2015a).

Our top model explaining bed site selection at the microsite level in winter supported thermoregulation over predator avoidance, although parameters in our top thermoregulation model likely provided a predator avoidance function as well. Thermoregulation may have been more important than predator avoidance in winter due to the cold temperatures in our study system (average winter temperature: −7.2 °C, with extreme temperatures of −36.7 °C, Gros Ventre SNOTEL weather station). Nevertheless, cougar beds were disproportionately found in our “rugged” habitat class, characterized by cliff bands and boulder fields. Rugged terrain features do provide thermoregulatory benefits, especially when southern-facing, but they also provide anti-predator benefits as well, as by definition, they are “escape terrain” (Akenson, Henjum & Craddock, 1996; Akenson et al., 2003).

In contrast, our analyses of bed site characteristics at the landscape level in summer supported predator avoidance over thermoregulation. Unlike bed selection in the winter, aspect was not a significant factor in summer bed site selection. Warmer summer temperatures may have precluded cougars from needing increased sun exposure to aide in body temperature regulation. Predator avoidance may also be more important in summer than winter for two additional reasons. First, bears wander the landscape in summer, and are well known to harass and steal food from cougars, as well as occasionally kill their kittens (Murphy et al., 1998; Ruth, 2004; Elbroch et al., 2015c). Second, the majority of cougar parturitions occur in this season, and the defense of kittens, which is primarily done through hiding them rather than active defense (Elbroch et al., 2015b), may influence cougar bed site selection at this time of year. At the microsite level, summer bed attributes reflected a combination of predator avoidance and thermoregulation. Cougars selected bed sites with high canopy cover that provided shade, high vegetative concealment to hide them from potential competitors, and rugged topography such as sloping hillsides and cliff bands.

Surprisingly, we did not find support for our hypothesis that cougars would bed in close proximity to “escape terrain.” Though the majority of bed sites in the both the summer (76.7%, *N* = 46) and the winter (90.0%, *N* = 72) were within 200 m of escape terrain, our analyses showed that cougars selected these features in proportion to their availability, at least as we measured this resource within our study. Alternatively, one could interpret our results to mean that escape terrain, as more broadly defined as habitat classes inclusive of cliffs and complex structures, is where cougars tend to live when sympatric with wolves (Ruth et al., 2011). Complex habitat structures are both advantageous to a cougar’s ambush hunting and predator avoidance strategies (Bryce, Wilmers & Williams, 2017). Given that fleeing competitors is energetically taxing (Bryce, Wilmers & Williams, 2017), cougar bed sites that mitigate predator avoidance may save cougars energy and increase their fitness.

Finally, our sample of cougars was relatively small; we examined bed sites from nine cougars at the landscape level and eight cougars at the microsite level. It will be important to examine bed site selection in other cougar populations with different competitor assemblages, as well as by other subordinate predators species, to ascertain the degree to which our findings are applicable to other systems.

## Conclusions

Our results suggest that cougar bed site selection facilitates both thermoregulatory and antipredator functions. The attributes of bed sites varied by season and order of selection, and were spatially explicit. As a subordinate predator, cougars appear to balance competing resource requirements with the risks associated with interacting with more dominant competitors. Because bed sites likely serve an anti-predator function, our research suggested that bed sites may provide a novel method by which researchers can measure competition refugia as part of habitat or home range selection for subordinate predators in multi-predator systems. Many researchers visit GPS clusters in the field to measure prey selection and predation rates (Knopff et al., 2010; Merrill et al., 2010), and accelerometer data is increasingly being applied to assign behaviors such as feeding or resting to GPS clusters that are not visited in the field (e.g., Fröhlich et al., 2012; Blecha & Alldredge, 2015). Therefore researchers can likely collect both microsite and landscape-level data for bed sites with minimal extra effort, and then utilize this information to further examine habitat selection among subordinate carnivore species, and how this might influence bioenergetics, interspecific competition, and ultimately, fitness.

##  Supplemental Information

10.7717/peerj.4010/supp-1 Supplemental Information 1 Descriptive analysis of winter bed sites at the microsite level We examined microsite characteristics of 80 winter bed sites from eight cougars. Here we provide the percentage of beds occurring in each attribute type: habitat type, topography, aspect, percent slope, percent canopy cover, percent concealment, ‘on feature’ (whether or not the bed was within 1 m of a prominent physical feature such as a tree or cliff band), the type of feature (if there was one), whether the bed was within 200 m of an escape terrain feature, the type of escape terrain feature that was present within 200 m, and the average distance to the nearest escape terrain feature.

10.7717/peerj.4010/supp-2 Supplemental Information 2 Descriptive analysis of summer bed sites at the microsite level We examined microsite characteristics of 60 summer bed sites from six cougars. Here we provide the percentage of beds occurring in each attribute type: habitat type, topography, aspect, percent slope, percent canopy cover, percent concealment, ‘on feature’ (whether or not the bed was within 1 m of a prominent physical feature such as a tree or cliff band), the type of feature (if there was one), whether the bed was within 200 m of an escape terrain feature, the type of escape terrain feature that was present within 200 m, and the average distance to the nearest escape terrain feature.

10.7717/peerj.4010/supp-3 Data S1 Supplementary data

## Additional Information and Declarations

### Field Study Permissions

The following information was supplied relating to field study approvals (i.e., approving body and any reference numbers): Our study was carried out on the Bridger-Teton National Forest (United States Forest Service, USFS Authorization ID JAC760804), Grand Teton National Park (NPS Permit GRTE-2012-SCI-0067), and National Elk Refuge (USFW permit NER12), with permission to handle cougars granted by the Wyoming Game and Fish Department (Permit 297).

### Data Availability

The following information was supplied regarding data availability: The raw data has been provided as Supplemental Files.
