# Supplementary material for: Bed site selection by a subordinate predator: an example with the cougar (Puma concolor) in the Greater Yellowstone Ecosystem
Source: PeerJ. 2017 Nov 14;5:e4010. doi: 10.7717/peerj.4010 (PMC5691788; doi:10.7717/peerj.4010)
Supplement: Supplemental Information 2 — We examined microsite characteristics of 60 summer bed sites from six cougars. Here we provide the percentage of beds occurring in each attribute type: habitat type, topography, aspect, percent slope, percent canopy cover, percent concealment, ‘on feature’ (whether or not the bed was within 1 m of a prominent physical feature such as a tree or cliff band), the type of feature (if there was one), whether the bed was within 200 m of an escape terrain feature, the type of escape terrain feature that was present within 200 m, and the average distance to the nearest escape terrain feature. [file peerj-05-4010-s002.docx]

**Descriptive analysis of summer bed sites at the microsite level. We examined microsite characteristics of 60 summer bed sites from six cougars. Here we provide the percentage of beds occurring in each attribute type: habitat type, topography, aspect, percent slope, percent canopy cover, percent concealment, ‘on feature’ (whether or not the bed was within 1 m of a prominent physical feature such as a tree or cliff band), the type of feature (if there was one), whether the bed was within 200 m of an escape terrain feature, the type of escape terrain feature that was present within 200 m, and the average distance to the nearest escape terrain feature.**

| Summertime beds | | | | | |
| --- | --- | --- | --- | --- | --- |
| Habitat Type | **Percent** | **N** | **On feature?** | **Percent** | **N** |
| Boulder field | 8.3% | 5 | Yes | 95% | 57 |
| Cliff band | 6.7% | 4 | No | 5% | 3 |
| Forest | 55.0% | 33 | **Feature Type** |  |  |
| Forest edge | 16.7% | 10 | Boulder | 3.5% | 2 |
| Meadow | 5.0% | 3 | Cave | 5.3% | 3 |
| Riparian | 5.0% | 3 | Cliff band | 7.0% | 4 |
| Sagebrush | 3.3% | 2 | Log jam | 17.5% | 10 |
|  |  |  | Tree or bush | 82.5% | 47 |
| Topography |  |  |  |  |  |
| Bench | 23.3% | 14 | **Near Escape Terrain?** |  |  |
| Cliff band | 8.3% | 5 | Yes | 76.7% | 46 |
| Flat | 6.7% | 4 | No | 23.3% | 14 |
| Drainage | 6.7% | 4 | **Terrain Type** |  |  |
| Ridgeline | 1.7% | 1 | None | 23.3% | 14 |
| Sloping hillside | 53.3% | 32 | Downfall | 45.0% | 27 |
|  |  |  | Cliff band | 16.7% | 10 |
| Aspect |  |  | Scree field | 15.0% | 9 |
| North | 36.7% | 22 | **Distance to terrain (m)** | **MEAN** | **SD** |
| South | 11.7% | 7 |  | 23.4m | 44.7 |
| East | 18.3% | 11 |  |  |  |
| West | 33.3% | 20 | **Canopy Cover (%)** | **MEAN** | **SD** |
|  |  |  |  | 91.7% | 17.2 |
| Slope (%) | **MEAN** | **SD** | **Concealment (%)** | **MEAN** | **SD** |
|  | 18.5 | 10.5 |  | 82.2% | 20.6 |
